# Investigating the nexus between authentic leadership, employees’ green creativity, and psychological environment: evidence from emerging economy

**DOI:** 10.1007/s11356-023-29928-1

**Published:** 2023-09-23

**Authors:** Fazal Ur Rehman, Ali Zeb

**Affiliations:** 1 Science and Research Centre, Faculty of Economics and Administration, University of Pardubice, Pardubice, Czech Republic; 2 Department of Business Management, Karakoram International University, Hunza Campus, Gilgit Baltistan, Pakistan

**Keywords:** Authentic leadership, Psychological environment, Employee’s green creativity

## Abstract

Employees’ green creativity is the basic input to organizational innovation capabilities, the prime focus of practitioners to stay competitive, and a mean to solve the society’s sustainable issues in dynamic markets. Hence, this study aims to evaluate the nexus between authentic leadership, psychological environment, and employees’ green creativity based on the theoretical lenses of social identity and social exchange theories that have rare application in these domains. Data were collected through questionnaires from 367 operational staff members of different technical training centers of renewable energy projects in Rawalpindi and Islamabad regions at Pakistan. The findings reveal that authentic leadership is a significant precursor of employee’s green creativity and self-efficacy. In addition, self-efficacy mediates while the environment of trust and safety has non-mediating role in the relationship between authentic leadership and employee’s green creativity. This work brings attention to the initiatives in technical training centers for renewable energy projects and contributes to the field of employees’ green creativity in the context of authentic leadership and psychological environment based on the philosophy of social identity and social exchange theories.

## Introduction

Green creativity enables employees to develop and implement environment genial practices in organizational settings and promotes the trend of sustainability. Green creativity leads to save production costs, gain competitive advantage, improve environmental efficiency, and add in the UN sustainability agenda 2030 (Zameer et al. 2021a, b; Rehman et al. 2023). In this regard, employees who contribute to creative ideas and innovation strategies can identify the opportunities to optimize resource utilization, reduce energy consumption, improve financial performance, and implement more efficient processes (Zameer et al. 2021a, b), perceived as the asset and key capability of an organization (Zeb et al. 2019; Imam et al. 2020). Due to these reasons, organizations often prefer to invest in the development of their employees to build sustainability-oriented capabilities (Zeb et al. 2019). In this consequence, authentic leadership is vital to influence individuals toward achieving the sustainable development goals (Zeb 2020).

Authentic leadership instigates the capacity to inspire and motivate subordinates, make sound decisions, and effectively manage resources and people, promoting the culture of green creativity (Cheng and Yang 2019; Zhou et al. 2018; Zameer et al. 2021a, b; Prokop et al. 2022), while the “employees’ green creativity is referred to as their potentials and roles to generate and initiate green innovative ideas” in the working environment (Amabile 1988). Authentic leadership encourages subordinates to share information, influence followers, share knowledge, and persuade a positive workplace environment (Ahmad et al. 2015; Wang et al. 2014; Xu et al. 2017) but needs further investigation in the context of employee’s green creativity. Usually, prior studies have focused on servant leadership, visionary leadership, transformational leadership to enhance creativity (Yoshida et al. 2014; Umalihayati et al. 2022; Nguyen et al. 2022; Zhou et al. 2018), but less focused on the role of authentic leadership (Zeb et al. 2019; Arici and Uysal 2022; Chaudhary & Panda 2018), especially in the employees’ green creativity in the perspective of psychological environment (Nasab and Afshari 2019; Sarkar 2019) in emerging economies. Studies are also enforced to determine the role of authentic leadership to explore the basic mechanisms to promote the culture of green creativity among firms (Sarkar 2019; Farrukh et al. 2023; Yasmeen et al. 2020; Zameer and Yasmeen 2022).

Moreover, authentic leadership promotes worker self-efficacy, enhance employee’s creativity and performance, creates flexible work environment, and improve organizational commitment (Meng et al. 2016; Bai et al. 2016; Laschinger et al. 2015). In particular, self-efficacy motivates the employees to engage in more innovative activities to foster green creativity (Yang et al. 2017; Nguyen et al. 2022; Maria et al. 2022), while the environment of trust is the “individual’ perception about workplace safety and the environment of reliance” which affects the creativity ability (Anderson and West 1998). Besides, prior studies (e.g., Semedo et al. 2018; Zeb et al. 2020) have directed to investigate the role of authentic leadership in various theoretical settings such as the mediating roles of self-efficacy and the environment of trust and safety to explore the relationship between authentic leadership and employees’ green creativity.

But till now, there is limited literature of these circumstances to explore employee’s green inventiveness (Ma et al. 2017), especially in growing markets (Zhou et al. 2018; Lee et al. 2019; Ribeiro et al. 2017; Wang et al. 2021a, b). Typically, earlier studies have investigated self-efficacy and the psychological environment in various contexts but never focused to examine the influence of self-efficacy and the environment of trust and safety on the employee’s green creativity in the technical training centers of renewable energy projects. Earlier studies also ignored so far to evaluate the mediating role of self-efficacy and the environment of trust and safety on the relationship between authentic leadership and the employee’s green creativity, exactly, in the technical training centers of renewable energy projects in emerging economies. Therefore, it is necessary to explain how a leader behaves to have an impact on the employees’ green inventiveness in the context of psychological environment. Hence, this study has the following shortcomings:

1. Does authentic leadership empower the environment of trust and safety?
2. Does authentic leadership encourage self-efficacy among employees?
3. What are the effects of the environment of trust and safety and self-efficacy on the employee’s green creativity?
4. Does authentic leadership enrich the employee’s green creativity?
5. Do the environment of trust and safety and self-efficacy among employees mediate between authentic leadership and the employee’s green creativity?

Consequently, to clarify the raised research questions and achieve the objectives, we embarked on a study to explore the relationship among the defined constructs. Hence, this study contributes by adapting the social identity and social exchange theory in the theoretical lens, considered the issues elevated by earlier scholars, and theorized the connection of leadership style by conceptualizing a model of employee’s green creativity. It also contributes by evaluating the mediation of the environment of trust and safety and self-efficacy between authentic leadership and the employee’s green creativity. Moreover, this study adds by investigating the application of authentic leadership in the technical training center of renewable energy projects in emerging economy and responding to the calls for further study on the association between authentic leadership and employees’ green creativity (Zeb et al. 2019). This study contributes by merging the literature of authentic leadership, psychological environment, and the employees’ green creativity based on the theoretical lens of social identity and social exchange theories. This study also contributes by validating the proposed research model based on the findings from emerging economy. However, the first part of this study involves by evaluating the existing literature, followed by the analysis of the connection between authentic leadership, psychological environment, and the employees’ green creativity. The concluding segment summarizes the findings, conclusions, and recommendations for future research.

## Literature review

Based on the philosophical background of social identity and social exchange theories, this study examines the association among authentic leadership, self-efficacy, the environment of trust and safety, and employees’ green creativity (Tajfel 1982). Social identity theory focuses to maintain the sense of positive distinctiveness among the group members (Tajfel and Turner 1986) while social exchange theory presumes that a social behavior occurs due to exchange process (Jonason and Middleton 2015). However, creativity is viewed as a cognitive and social process as well as a personality trait, rather than just a product of individual talents and traits (Amabile and Pillemer 2012). The study operationalized social categorization by explaining the nexus between authentic leadership and green creativity based on the environment of trust and safety, as well as self-efficacy of employees. Authentic leadership braces a person’s positive attitude and actions as well as strives for open and honest relationships with followers along with several favorable outcomes (Ogunyemi and Ogunyemi 2020; Gardner et al. 2021). Therefore, the social identity and social exchange theories are applied to evaluate the role of positive senses and social behavior of leaders in influencing the employee’s green creativity and their psychological mindset.

### Theory and hypothesis development

Authentic leadership is considered as a multifaceted construct that includes internal moral perspective, relational transparency, balanced processing, and leader self-awareness. It refers to the degree to which the leader is conscious of exhibiting a pattern of openness and clarity and is consistent in disclosing and acting upon personal principles, sentiments, and intentions (Walumbwa et al. 2008). According to Semedo et al. (2016), firms can put more emphasis on hiring leaders and supervisors who have value transparency and self-awareness in their interactions with employees. The study demonstrated that the leaders and supervisors can process internal moral and balance processing information to produce the desired outcomes both at the organizational and individual levels. Semedo et al. (2018) discovered that authentic leadership affects individual’s affective commitment and their inventiveness. The study emphasized that authenticity boosts creativity and worker’s capacity to deal with changing obstacles at workplace. Likewise, George (2003) stressed that authentic leadership improves the relationship between leaders and their teams and enhances ecological efficiency, which in turn leads toward positive attitudes and behaviors, that can result in highly green creativity (Rod and Ashill 2009; Regoet al. 2012; Walumbwa et al. 2011; Karman et al. 2023). Based on the assumption that effective association between employees and managers may improve employees’ views toward overall workplace initiatives, this study takes a similar approach to the leader-member interaction. As a result, we put forward the following hypothesis:

- **H1.** Authentic leadership enhances employee’s green creativity.

The environment of trust is related to how the group members perceive these two aspects: how trust-related issues are handled and whether or not an employee’s behavior is consistent with the characteristics of trust (Costigan et al. 1998). According to Luthans and Avolio (2003), authentic leadership helps to foster a healthy atmosphere that is centered on trust, compassion, and a culture that values and encourages ethical behavior. By establishing cooperative relationship networks and environment of justice and encouraging people to share their independent views, authentic leaders can quickly gain the respect and trust of their workforce (Zeb et al. 2021; Batmomolin et al. 2022). Furthermore, Fransen et al. (2011) emphasized that achieving a reliable mode of activity within the team environment requires member’s participation that engenders a strong perception of mutual trust among employees by using the behavioral approach of authentic leaders. Avolio et al. (2004) praised that authentic leaders are transparent in their interpersonal ties with subordinates/members and share their true beliefs, values, and feelings. Authentic leadership behavior is characterized by a highly developed organizational context as well as positive emotional abilities. These capabilities promote constructive conduct both in the leaders and subordinates which furthers improve their personal growth. According to Gonçalves and Brandão (2017), the psychological capital and safety of the group are predicated by the leader humility. However, prior studies have evaluated the association of authentic leadership with various factors in organizational settings but less focused to examine the role of authentic leadership with the environment of trust and safety. Therefore, we proposed the following hypothesis:

- **H2.** Authentic leadership enhances the environment of trust and safety.

Self-efficacy of the employees is a motivational tool which stimulates their risk-taking propensity in difficult situations, taking decisions, and participating in different work activities (Sharif and Raza 2017; Khalil and Siddiqui 2019; Rehman et al. 2018). The level of self-efficacy is differing among individual and is affected by many factors. However, authentic leaders are not only aware of their own strengths and limitations but also aware of their subordinates. It is their confidence, which emphasizes the employee’s strength and empowers them to overcome their deficiencies that enable the employees to acquire a high-level self-efficacy. Therefore, leaders can maintain an open and honest relationship with their staff to motivate them and increase their self-efficacy to optimize the level of creativity. One of the dimensions of psychological capital is self-efficacy, and it may be argued that authentic leadership affects self-efficacy (Avolio and Luthans 2006). Hence, we proposed the following hypothesis.

- **H3.** Authentic leadership enhances self-efficacy among employees.

Bock et al. (2005) stated that mutual trust among group members is a helpful mechanism for organization to handle tasks outside the scope of their formal duty. Group members also share mutual opinions regarding problems from various angles of knowledge. Amabile (1988), Chaudhry et al. (2023), and Zeb et al. (2019) argued that a trustful environment encourages employees’ creativity and safety. These studies further pointed out that the mutual trust and support among group members can enhance the confidence in their conducts and lead them to apply new tactics in their job. Zhou and George (2003) noted that the trust can improve interactions and cooperation among members, thereby instigating creativity and innovation. However, it is compulsory that supervisors should help in making creative ideas and devising an environment of trust to improve individual creativity (Tsai et al. 2015). Hsu et al. (2007) and Zameer et al. (2021a, b) also opined that importance of a strong atmosphere of mutual trust among group members could not be underestimated because it leads to creativity. Moreover, safety is also a significant predictor of employees’ creativity. Baer and Frese (2003) focused on safety practices, employees’ creativity, and performance. The study observed that in safe and protected environment, employees feel less interpersonal pressure and used their full strength for creativity. This leads to the following hypothesis:

- **H4.** Environment of trust and safety enhances employees’ green creativity.

“Self-efficacy is an individual’s belief that can organize and complete specific tasks” (Bandura 1994). Tierney and Farmer (2002) also presumed that “self-efficacy refers to employee belief in their own ability to be creative in their job roles.” Job self-efficacy, job tenure, job complexity, and leader role contribute to the strength of creative efficacy beliefs. Tierney and Farmer (2002, 2011), Kim et al. (2019), and Han and Bai (2020) claimed that self-efficacy predicts creative performance beyond the predictive effects of job self-efficacy. Several theoretical and empirical studies supported the evidence that self-efficacy improves employees’ creativity. Based on this discussion, we propose the following hypothesis:

- **H5.** Self-efficacy enhances employee’s green creativity.

Based on the contingency approach, psychological environment of trust and safety was used as a mediating concept. Zeb et al. (2019) proved that authentic leadership develops a positive environment of trust and safety through the authenticity to stimulate innovative skills. The same was concluded by Meng et al. (2016) where trust and safety mediated the links between authentic leadership and employee’s creativity. A similar study in Taiwanese firms also found that authentic leadership and employees’ creativity are mediated through leader-member exchange and a safe psychological environment (Xu et al. 2017). Xu et al. (2017) used leader-member exchange (LMX) approach between authentic leadership and creativity and found that employee’s perception toward authentic leadership enhanced the psychological trust and safety through LMX relationships, which further influence the level of creativity. Therefore, the current study looked at how safety and trust act as linking factors in the relationship between authentic leadership and employees’ green creativity. Based on the contingency approach, authentic leadership is the antecedent concept and deliberates the mediating effect of psychological environment of trust and safety and employees’ creativity. Therefore, we propose the following hypothesis:

- **H6.** Environment of trust and safety mediates the link between authentic leadership and employees’ green creativity.

Literature on the topic highlighted that leader role has an important effect on the employee’s creativity and proposed a notion that self-efficacy mediates the relationship between leader role and creativity. Evidently, Jaiswal and Dhar (2015), Santoso et al. (2019), and Azim et al. (2019) revealed a positive relationship between transformational leadership, self-efficacy, and creativity. The study of Yang et al. (2017) also found that self-efficacy mediates the link between servant leadership and employee’s creativity. The study further concluded that servant leadership predicted self-efficacy, which in turn promotes the employees’ creativity. Zeb et al. (2019) opined on intrinsic motivation principle of creativity and confirmed that authentic leadership stimulates creative beliefs promoting employees’ creativity. However, prior studies have evaluated the mediating role of self-efficacy in various context, but less focused to examine the mediating role of self-efficacy on the relationship between authentic leadership and the employee’s green creativity in the technical training centers of renewable energy projects. Therefore, we propose the following hypothesis:

- **H7.** Self-efficacy mediates the link between authentic leadership and employees’ green creativity.

### Conceptual framework

**Fig. 1:**
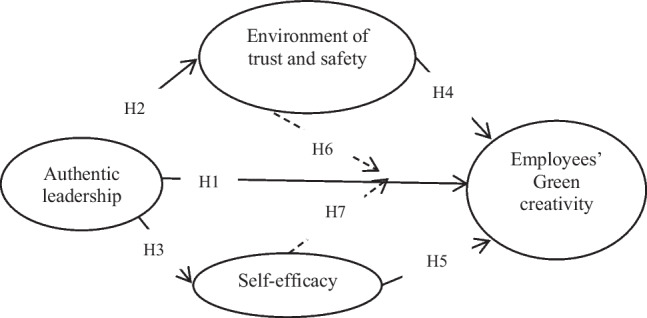
Conceptual model of the study

This study revolves around the idea of green creativity, which refers to the ability of employees to develop and implement environment friendly practices and initiatives within organizations. Green creativity is seen as a key capability for organizations to stay competitive in today’s dynamic environment around the world. However, this study integrates the concept of authentic leadership, psychological environment, and the employee’s green creativity to visualize the conceptual framework for the operationalization of study as shown in (Fig. 1).

## Method

### Sample and procedures

Operational staff members of different technical training centers of renewable energy projects at Rawalpindi and Islamabad regions were the population of this research study. First, we got an approval from the CEO/concerned officers about data collections from the operational staff members of the technical training centers of renewable energy projects. The technical training center of renewable energy projects was the subject of the study because of its actual and potential contributions to public sector. These technical training centers for renewable energy projects decrease the ratio of unemployment, handle the energy issues, and focus on the UN sustainability agenda 2030.

A non-probability, convenience sampling technique was applied followed by the recommendations of Zeb et al. (2021) . This type of sampling technique allows to quickly and easily gather data as they can select participants who are readily available and accessible. This is a useful approach when there is limitation of resources and time and suitable in industrial settings (Baxter et al. 2015; Zeb et al. 2019; Rasool et al. 2023). Therefore, this study preferred to collect data through convenience sampling techniques due to the limitation of resources and time and unstructured industrial settings.

In primary research, usually the questionnaire-based survey is applied to obtain the views of respondents to predict the relationship among construct (Zameer et al. 2021a, b). However, on the defined scale in this study, all the responses were recorded through five-point Likert scale ranging from strongly disagree = 1 to strongly agree = 5. Before the distribution of the survey forms, respondents were guided regarding the aims of the study and questionnaires. A total of 450 survey forms were distributed, but only 367 (81.56% response rate) questionnaires were received in complete form. The data is being truly used for the first time, presenting the current dynamics of authentic leadership, psychological environment of trust and safety, and the employees’ green creativity of the technical training centers of renewable energy projects in Rawalpindi and Islamabad regions.

### Demographic profile

The results of the respondents (please refer to Table 1) indicated that 345 (94.01%) were male among the respondents and 22 (5.99%) were female and 101 (27.53%) were < 20–29 years old, 118 (32.16%) were 30–39 years old, and 148 (40.33%) were 40–49 years old. The results of the respondents’ education indicated that 103 (28.07%) have SSC level of education, 144 (39.23%) have HSC level of education, 75 (20.44%) have BA level of education, and 45 (12.27%) have master and MS level of education. The results of the respondent’s experience indicated that 159 (43.33%) have < 10–19 years of experience, 146 (39.79%) have 20–29 years of experience, and 62 (16.90%) have 30–39 years of experience, respectively.

**Table 1:** Socio-demographic details of the respondents

Demographic characteristics	Frequency	Percentage
Gender		
Male	345	94.01
Female	22	5.99
Age		
< 20–29 years	101	27.53
30–39	118	32.16
40–49	148	40.33
Education		
SSC	103	28.07
HSC	144	39.23
BA	75	20.44
Master/MS	45	12.27
Experience		
< 10–19	159	43.33
20–29	146	39.79
30–39	62	16.90

### Measures

Authentic leadership was measured by a six-item scale, e.g., one sample items “my immediate leader is able to improve his/her interactions with others by soliciting feedback.” The scale was taken from the study of Walumbwa et al. (2008) that have a valid Cronbach’s alpha value 0.827. Environment of trust and safety was measured by a six-item scale, e.g., two sample items “I trust my co-workers” and “I am confident that I can handle the physical demands at work.” The scale was taken from the study of Kahn (1990) and May et al. (2004) having valid Cronbach’s alpha values 0.827 and 0.910. Self-efficacy was measured with a six-item scale, e.g., one sample item “I will be able to achieve most of the goals that I have set for myself.” The scale was taken from the studies of Igbaria and Guimaraes (1993) and Rehman et al. (2021a, b) having a valid alpha value 0.86. Employee’s creativity was also measured by a six-item scale, e.g., one sample items “suggests new ways to achieve goals or objectives.” The scale was taken from the study of Zhou and George (2003) having valid Cronbach’s alpha values 0.877.

### Technique of analysis

The study analyzed the collected data by using the technique of partial least squares structural equation modeling (PLS-SEM). PLS-SEM is preferable to analyze the complex models (where there are no issues of small sample size), test the conjectures, and measure the validity of theory; is highly recommended in formative style of theoretical consideration; and is preferable in predictive studies (Raza et al. 2021; Rehman et al. 2022; Raza and Khan 2022). The collected data were examined by employing a bootstrapping procedure with the sample that consists of 5000 subsamples (Hair et al. 2012; Raza et al. 2020). PLS-SEM justifies the association in an organized way and displays the results in a single click (Rehman et al. 2021a, b). Hence, PLS-SEM was the more suitable option to establish association among the defined constructs.

## Results

### Measurement model

**Fig. 2:**
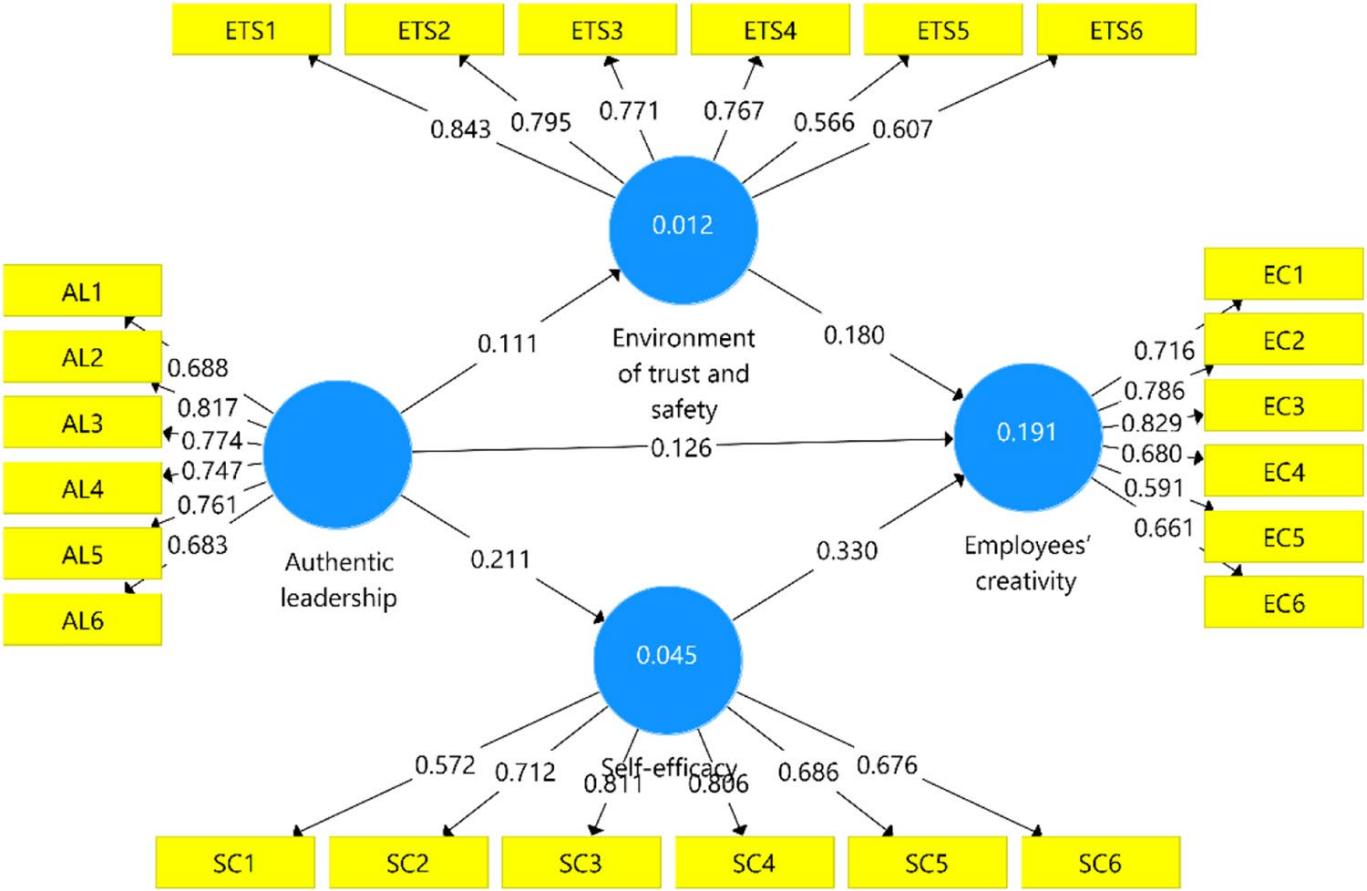
Measurement model of the study

This exploratory study aims to establish relationship among the defined constructs; therefore, PLS-SEM was selected to analyze the research model. The PLS-SEM procedure usually comprises two sub-models: the measurement model and the structural model (Rehman and Zeb 2022; Rehman et al. 2021a, b). Before assessing the path relationships of the model, the validity and reliability of various constructs of the measurement model were examined to gauge whether the study’s constructs are valid and reliable or not. To ensure this, the first step was to examine the construct reliability and content validity. As content validity concerns with the reliability of the constructs, according to Hulland (1999), content validity and reliability are assessed by examining the loadings of respective items on their respective latent construct. A study recommended that the standardized loading estimates should be equal to or greater than 0.5, and the ideal range is 0.7 or higher (Rehman 2023). The alpha values of all constructs were greater than 0.7, which signifies that all constructs were reliable (Rehman and Prokop 2023). All constructs of outer loadings ranging from 0.566 to 0.843 were considered acceptable as they are above the proposed criteria of 0.55 (Raza et al. 2020; Raza and  Khan 2022). The composite reliability values of all constructs ranging from 0.861 to 0883 were greater than 0.7, as per the recommended criteria (Hair et al. 2012) that it should be greater than 0.7. The AVE values of all constructs ranging from 0.511 to 0.575 were greater than 0.5 thresholds. However, the measurement model values signified high level of internal consistencies. The results of the measurement model are shown in Table 2 and Fig. 2, respectively.

**Table 2:** Results of the measurement model

Constructs	Items	Factor loadings	Alpha value	AVE	C.R
Authentic leadership			0.840	0.557	0.883
	AL1	0.688			
	AL2	0.817			
	AL3	0.774			
	AL4	0.747			
	AL5	0.761			
	AL6	0.683			
Environment of trust and safety			0.832	0.535	0.871
	ETS1	0.843			
	ETS2	0.795			
	ETS3	0.771			
	ETS4	0.767			
	ETS5	0.566			
	ETS6	0.607			
Self-efficacy			0.805	0.511	0.861
	SC1	0.572			
	SC2	0.712			
	SC3	0.811			
	SC4	0.806			
	SC5	0.686			
	SC6	0.676			
Employees’ creativity			0.814	0.511	0.861
	EC1	0.716			
	EC2	0.786			
	EC3	0.829			
	EC4	0.680			
	EC5	0.591			
	EC6	0.661

### Discriminant validity

The outcome of the measurement model shows that there are no issues of multicollinearity (please refer to Table 3) as per the procedure of prior studies (Rehman 2023), because the square root of AVE for each construct is greater than the correlation of other latent constructs, which implies adequate discriminant validity (Fornell and Larcker 1981).

**Table 3:** Discriminant validity of the constructs

Constructs	Fornell-Larcker criterion			
	1	2	3	4
1 Authentic leadership	**0.746**			
2 Employees’ creativity	0.216	**0.715**		
3 Environment of trust and safety	0.111	0.223	**0.732**	
4 Self-efficacy	0.211	0.373	0.089	**0.715**

Values in Bold Indicate diagonal values

### *R*^*2*^coefficient of determination

The path coefficient value *R*^2^ shows that only 19.1% of the total variance was explained by authentic leadership, environment of trust and safety, and self-efficacy. Furthermore, *R*^2^ shows that 1.2% and 4.5% of the total variance in environment of trust and safety and self-efficacy were explained by authentic leadership. Further, the VIF values, 1.056, 1.000, and 1.000, were less than 5 which is an acceptable range (please refer to Table 4) as per the criteria of the previous studies (Rehman et al. 2023).

**Table 4:** Coefficient of determination *R*^2^ variance inflation factor

Constructs	*R*^2^	VIF
Employees’ creativity	0.191	1.056
Environment of trust and safety	0.012	1.000
Self-efficacy	0.045	1.000

### Structural path analysis

The detailed description of results of the structure model was presented (please refer to Table 5). Authentic leadership has significant impacts on the employee’s green creativity (*β* = 0.126, *t* = 2.958, *p* = 0.000) and supports H1. On the other side, authentic leadership has insignificant impacts on the environment of trust and safety (*β* = 0.111, *t* = 0.456, *p* = 0.146) and rejects H2. Authentic leadership has also significant impacts on self-efficacy (*β* = 0.211, *t* = 3.809, *p* = 0.000) and supports H3. Moreover, the environment of trust and safety has also significant impacts on the employee’s green creativity (*β* = 0.180, *t* = 2.373, *p* = 0.018) and supports H4. Furthermore, self-efficacy has also significant impacts on the employees’ green creativity and supports H5. The indirect structural path values (*β* = 0.020, *t* = 1.160, *p* = 0.247) show that the environment of trust and safety did not mediate the link between authentic leadership and the employee’s green creativity and rejects H6, while the indirect structural path values (*β* = 0.070, *t* = 3.069, *p* = 0.002) show that self-efficacy significantly mediates the link between authentic leadership and the employee’s green creativity and supports H7.

**Table 5:** Results of the structural model

No. H	Relationships	*β* values	*t* values	*p* values	Decision
H1	Authentic leadership → employees’ creativity	0.126	2.958	0.000	Accepted
H2	Authentic leadership → environment of trust and safety	0.111	1.456	0.146	Rejected
H3	Authentic leadership → self-efficacy	0.211	3.809	0.000	Accepted
H4	Environment of trust and safety → employees’ creativity	0.180	2.373	0.018	Accepted
H5	Self-efficacy → employees’ creativity	0.330	6.686	0.000	Accepted
H6	Authentic leadership → environment of trust and safety → employees’ creativity	0.020	1.160	0.247	Rejected
H7	Authentic leadership → self-efficacy → employees’ creativity	0.070	3.069	0.002	Accepted

## Discussion

The first pathway of this study illuminates the direct effect of authentic leadership on the employees’ green creativity, while the second pathway delineates the mediating effect of the environment of trust and safety and self-efficacy between authentic leadership and employee’s green creativity. The result of the first pathway articulates that authentic leadership positively enhanced employees’ green creativity. However, the findings of the first pathway are consistent with the prior research studies of Cerne et al. (2013), Liqun et al. (2017), Semedo et al. (2018), Zeb et al. (2019), and Imam et al. (2020). In the second pathway, self-efficacy partially mediated the relationship while environment of trust and safety did not mediate the relationships between authentic leadership and the employees’ green creativity. The results supported that higher authenticity of the leaders can engage employees in green creative activities. When employees feel that our leader is authentic and able to provide a creative environment, then they will habitually reciprocate green creativity in the workplace. The findings of our study are partially supported by the previous research of the following authors: Amabile et al. (1994), Zeb et al. (2019), and Phuong and Takahashi (2021). The studies stated that authentic leadership developed truthful relationships with the subordinates, enhancing motivation and promoting innovative work environments and fostering employee’s creativity. Our findings are also contrary to the study of Malodia and Goyal (2023) who found the sequential mediation effect of workplace inclusion and employee engagement in the relationship between authentic leadership and employee’s creativity in the IT sector. Our study contributed to the body of knowledge by using the psychological environment of trust and safety and self-efficacy as mediating mechanism between the authentic leadership and employee’s green creativity in the technical training sector of renewable energy projects.

The results confirm that authentic leadership has a strong association with the employees’ green creativity in the technical training centers of renewable energy projects at Rawalpindi and Islamabad regions. Amabile et al. (1994) supported the view that perceived leadership support has a positive influence on the employee’s green creativity. Phuong and Takahashi (2021) support the results of this study and presumed that the perceived leadership support has positive impacts on the employee’s creativity. The study further stated that authentic leadership acts as a role model to inspire employees. The direct effect of authentic leadership on self-efficacy and employees’ creativity is also confirmed by the previous research studies of Lee et al. (2019), Lei et al. (2021), Niswaty et al. (2021), and Javed et al. (2021).

A study that has been conducted by Sumanth et al. (2023) stated that authentic leadership and LMX approach foster employees’ proactive orientation and creativity with the theoretical lens of social identity and social exchange theories at Central Europe and the USA. We provide a theoretical lens of both theories to assess a multilevel model of creativity and authentic leadership theory. We also concluded from the results that authentic leadership style promotes green creativity of the employees.

Furthermore, this study also clarified that the environment of trust and safety did not mediate the relationship while self-efficacy mediated the relationship between authentic leadership and the employees’ green creativity. These findings contribute to the study of Yang et al. (2017) who called for additional research to check the effect of leaders on creativity on a multi-mediation level. Fewer studies by Yang et al. (2017), Wang et al. (2021a, b), and Ma et al. (2021) explored the impact of authentic leadership on the environment of trust and safety and self-efficacy and employee’s creativity. Yoshida et al. (2014), Yang et al. (2017), and Xu et al. (2017) called for further research to collect data from different organizations and to examine the effect of leaders at multi-mediation levels. The responses collected from technical training center employees of renewable energy projects at Rawalpindi and Islamabad regions clarified that authentic leadership instigates employees’ self-efficacy as well as the environment of trust and safety, which boost green creative ideas in the workplace.

### Conclusion

In conclusion, results of the study indicated that authentic leadership positively influences employees’ green creativity and supports previous research findings. The analysis also revealed that self-efficacy partially mediated the relationship between authentic leadership and green creativity, while trust and safety in the environment have non-mediating effect. These findings suggest that when leaders demonstrate authenticity and create a supportive and creative environment, then employees are more likely to engage in green creative activities. The results also align with the prior research and emphasized on the importance of authentic leadership relationships in fostering employee’s creativity (Malodia and Goyal 2023). Additionally, the study contributes to the theoretical lens of social identity and social exchange theories in assessing the relationship between authentic leadership and employee’s green creativity. Overall, this research provides valuable insights into the role of authentic leadership in promoting employees’ green creativity and highlights the need for further investigation into the multi-mediation effects of leaders on creativity.

### Theoretical implication

The study contributes to the existing theoretical knowledge by highlighting the role of authentic leadership in influencing employees’ green creativity in the light of social identity and social exchange theories. It supports the notion that authentic leadership plays a significant role in fostering green creativity among employees in the context of renewable energy projects. The study builds upon prior empirical studies that have already established a linkage between authentic leadership and creativity. By reinforcing these findings in the specific context of a technical training center for renewable energy projects in Rawalpindi and Islamabad regions at Pakistan, the study strengthens the theoretical understanding of the relationship between authentic leadership and green creativity among employees in the light of self-efficacy and the environment of trust and safety in emerging markets.

### Managerial implication

The study suggests that the management or leadership of the technical training center of renewable energy projects in Rawalpindi and Islamabad regions, Pakistan, should create a counter-productive environment that encourages green creativity among employees. This managerial insight emphasizes the importance of providing an atmosphere that fosters green creativity and supports employees’ efforts in contributing toward green initiatives. The study highlights the role of authentic leaders in improving employees’ output and moral levels. It suggests that leaders should act as the role models and set the norms and standards for employees to be followed. By recognizing the significance of authentic leadership in influencing employees’ counter-productive green behavior, the study provides guidance for managerial practices that can enhance organizational outcomes. The study emphasizes on the importance to create a psychologically safe and trusted environment within the organization. It suggests that the management or leadership should foster the atmosphere where employees feel free of fear and risks, enabling them to actively share their knowledge within organization and contribute toward green creativity. This managerial recommendation promotes collaboration, building trust, and promotes knowledge sharing culture among employees.

### Limitations and further research

This research was the first such type of empirical attempt to assess the links between authentic leadership, environment of trust and safety, self-efficacy, and the employee’s green creativity in the context of technical training centers of renewable energy projects at Rawalpindi and Islamabad regions of Pakistan. This study provides the potential directions for future research due to some major limitations. Firstly, one of the limitations is the sample data, which is only concern with technical training centers at Rawalpindi and Islamabad regions. Hence, the results of this study are limited to technical training centers of renewable energy projects. Therefore, the results of this study posses’ generalizability challenges. Secondly, this study has collected data from the technical training center industry employees and assessed authentic leaderships and employee’s green creativity. Future studies can explore the employee’s green creativity by collecting data from other broad level of organizations in another managerial context. Thirdly, in this study, we used a multidimensional mediation of self-efficacy and the environment of trust and safety between authentic leadership and employee’s green creativity, while future studies can examine the role of gender, education, and age in the capacity of control variables to further enrich the relevant knowledge area. Hughes et al. (2018) argued that the future research may assess identification of different mechanisms and concept that may offer supplementary concepts in understanding the relationship between leader and subordinates’ relationships. Fourthly, this study used a cross-sectional research approach. Future studies can use longitudinal research approach to assess the association between authentic leadership and the employee’s green creativity.

## Data Availability

Data will be provided on the request to the corresponding author.
